# M to T Rearrangement: An Approach to Correct Webbed Neck Deformity

**DOI:** 10.1155/2014/682806

**Published:** 2014-01-09

**Authors:** Ananth S. Murthy, Margeaux McGraw

**Affiliations:** ^1^Division of Plastic Surgery, Akron Children's Hospital, One Perkins Square, Akron, OH 44308, USA; ^2^The University of Toledo College of Medicine, Toledo, OH 43606, USA

## Abstract

For the Noonan syndrome patient, the most concerning physical defect is often congenital webbing of the neck or pterygium colli. We present a patient with pterygium colli and a low and laterally displaced nuchal hairline. Since its description, various surgical approaches have been implemented to correct the deformity. Previously reported posterior and lateral approaches have notable disadvantages with regard to hairline displacement and recurrence. In order to address these disadvantages, a new surgical technique was used on this patient. We have termed this technique an M to T rearrangement. Using a lateral approach, the M and T incisions are made and the trapezial fascial web is directly visualized and able to be completely excised. This prevents the recurrence seen with the use of posterior techniques. Inferolateral displacement of hair-bearing skin can be removed with resection of the superior intervening triangle and improves the appearance of the low nuchal hairline. The excision of excess skin along with the zig-zag closure also prevents postoperative scar contraction and recurrence. An important effect of this technique is the prevention of anterior displacement of hair bearing skin. M to T rearrangement is an effective technique for the correction of webbed neck deformities seen in Noonan and Turner syndromes.

## 1. Introduction

A syndrome involving valvular pulmonary stenosis, small stature, hypertelorism, mild mental retardation, undescended testes, and skeletal abnormalities was first described by Noonan and Ehmke in 1963 [1]. Noonan syndrome is characterized by short stature, congenital heart defect, and developmental delay of variable degree. Other findings can include broad or webbed neck, pectus deformity, cryptorchidism, characteristic facies, varied coagulation defects, lymphatic dysplasias, and ocular abnormalities [2]. The syndrome is fairly common, as the incidence is estimated to be approximately 1 : 1,000–2,500 live births [3]. An autosomal dominant pattern of inheritance has been determined and attributed to missense mutations in the PTPN11 gene on chromosome 12 in 50% of cases [4].

For the Noonan syndrome patient, the most concerning physical defect is often congenital webbing of the neck or pterygium colli. Kobylinski initially reported this deformity in 1883 and the name “pterygium colli” was coined by Funke in 1902 [5, 6]. Pterygium colli involves an ectopic fibrotic facial band superficial to the trapezius muscle. Excess hair-bearing skin is also present and extends down the cervical region well beyond the normal hairline.

Since its description, various surgical approaches have been implemented to correct the deformity. Previously reported posterior and lateral approaches have notable disadvantages with regard to hairline displacement and recurrence. In order to address these disadvantages, a novel surgical technique was used on this patient. We have termed this technique an M to T rearrangement.

## 2. Case Report

A 13-year-old male was evaluated for congenital webbing of the neck secondary to Noonan syndrome. The patient presented with characteristic features of pterygium colli with a low and laterally displaced nuchal hairline. Presurgical testing did not reveal any coagulation abnormalities. These patients can often have specific factor deficiency or platelet aggregation anomalies, but none were noted with this patient. Surgical treatment was outlined to address both the neck and hairline abnormalities.

**Figure 1:**
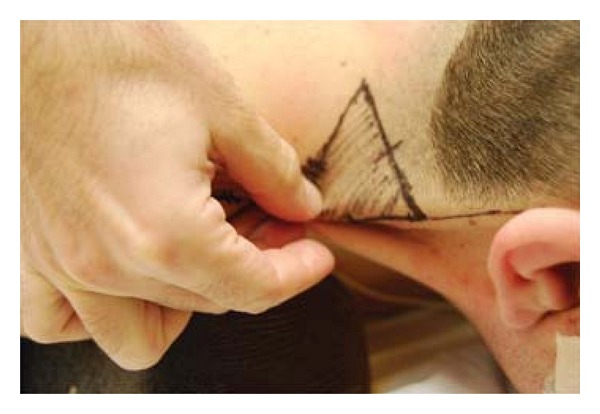
A pinch test of the skin overlying the trapezius muscle was used to determine the excess.

Under general anesthesia, the patient was placed in a prone position and the webbing was carefully marked from the mastoid to the acromion. A pinch test of the skin overlying the trapezius muscle was used to determine the excess (Figure 1). A horizontal incision was marked perpendicular to the web to form a “T.” This incision was placed parallel to a crease in the anterolateral neck. The length of this limb (releasing incision) was determined from the midpoint of the web line to the palpated edge of the pinch test.

**Figure 2:**
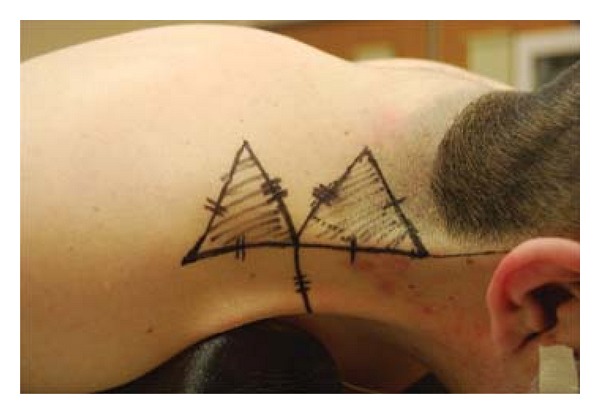
Markings for an M-T rearrangement. An incision is marked perpendicular to the web to form a “T.” This incision is parallel to a crease in the anterolateral neck. The length of this incision is determined by the pinch test. Then, a triangular flap whose apex is at the intersection of the limbs of the “T” is marked. This is an equilateral triangle, with the limbs and base of the triangle measuring the same length as the releasing incision. The base of the triangular flap is then connected to the superior and inferior edges of the webbing to form an “M.” Note that the inferolateral displacement of hair-bearing skin is present within the superior triangle.

A triangular flap whose apex was at the intersection of the limbs of the “T” was then marked. An equilateral triangle, with the limbs and base of the triangle measuring the same length as the releasing incision, was marked. It is paramount to orient the triangular flap in such a way so to exclude any hair-bearing skin that is commonly present in a patient with inferolateral displacement of the hairline. The base of the triangular flap is then connected to the superior and inferior edges of the webbing to form an “M” (Figure 2).

**Figure 3:**
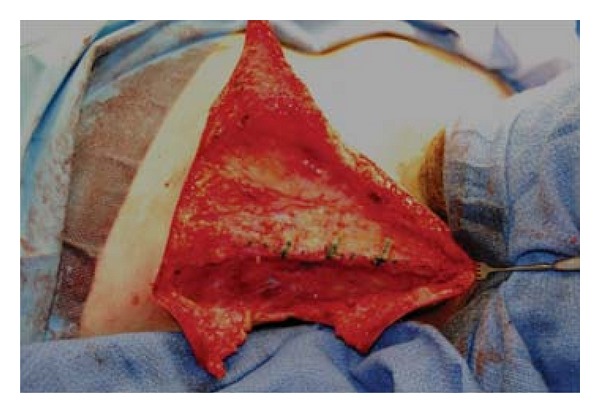
A lateral approach allows for complete visualization of fibrotic fascial band on the trapezius muscle. Markings indicate regions to be resected.

**Figure 4:**
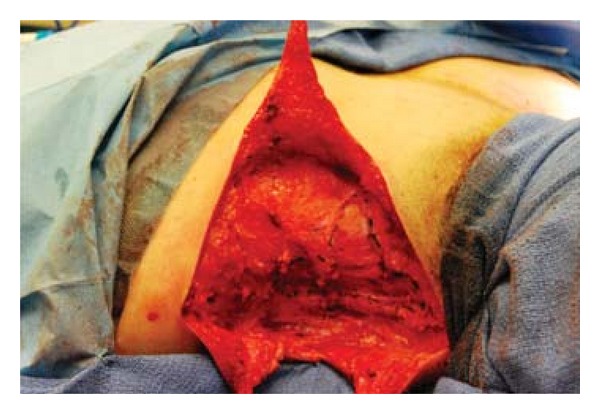
Fibrotic fascial band is excised and trapezial webbing is effectively treated.

The M and T incisions were made along the markings. The skin and subcutaneous tissue of the two intervening triangles that are present superior and inferior to the triangular flap were resected. The superior triangle usually incorporates the inferior and lateral displaced hairline and hair bearing skin. Then the fibrotic fascial band of the trapezius is fully visualized (Figure 3). The most important part of the procedure is to perform a complete excision of the trapezial fascial web in order to prevent recurrence. This effectively lengthened the trapezius and improved the neck profile (Figure 4). Meticulous dissection is required to visualize the spinal accessory nerve and protect it from injury.

**Figure 5:**
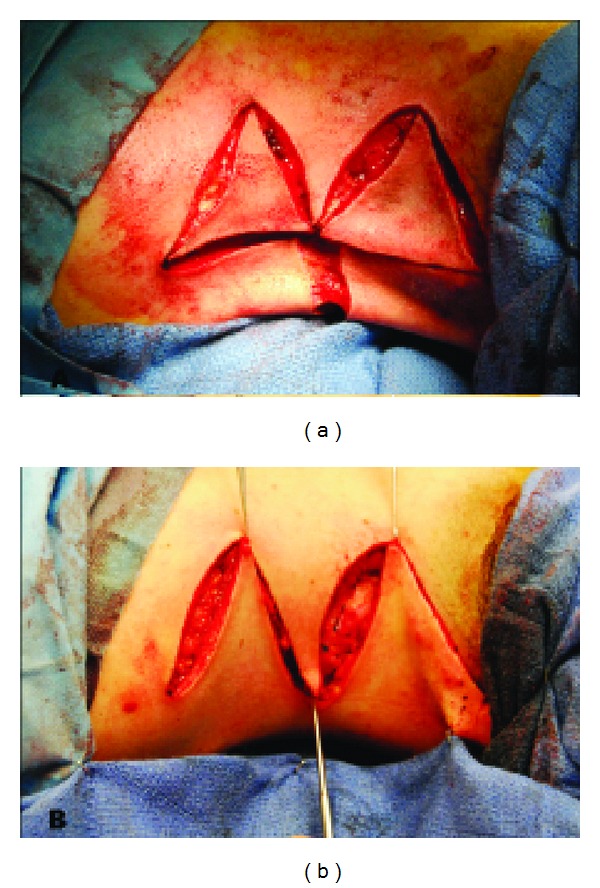
(a) Initial incisions made with hair-bearing skin to be removed within the superior intervening triangle; (b) completed zig-zag closure.

**Figure 6:**
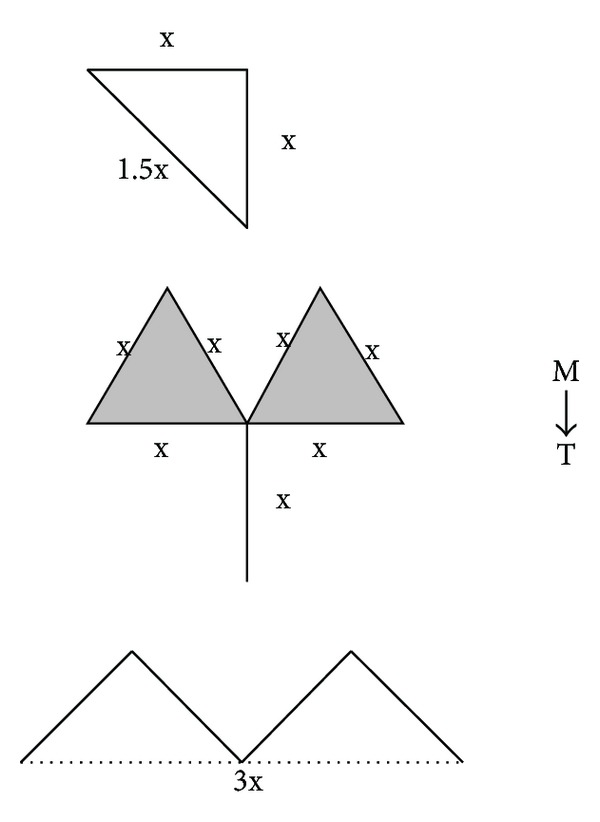
A diagrammatic view of the M to T rearrangement depicting the effective lengthening needed to treat the webbing.

The triangular flap is then advanced into the releasing incision in the neck. The remaining portions of the incisions fall into place to form a zig-zag closure, which in turn not only increases the incisional length, but also reduces the chances of recurrence (Figures 5 and 6).

**Figure 7:**
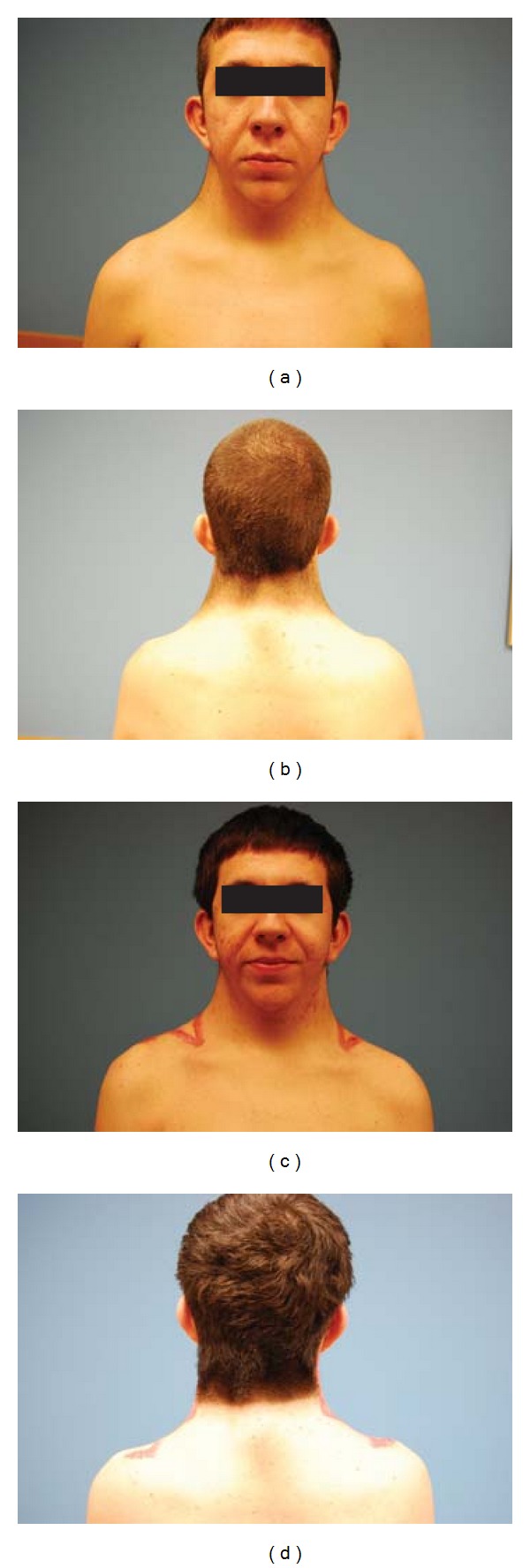
(a) Preoperative appearance demonstrating webbed neck deformity of Noonan syndrome; (b) preoperative low and laterally displaced nuchal hairline; (c) anterior appearance one year after surgical correction of pterygium colli with no evidence of anterior displacement of hair-bearing skin; (d) posterior appearance one year after surgical correction of pterygium colli with improved neck contour, improved nuchal hairline, and without evidence of recurrence.

Postoperatively, the incisions healed well and trapezius function was preserved. The hairline was improved by resection of hair-bearing skin within the superior intervening triangle. No recurrence was noted at the one-year followup (Figure 7). The patient did have hypertrophic scarring throughout both incisions. At the one-year followup, scar revision was offered. Even with the presence of hypertrophic scarring, the patient was quite content with the result and declined this option.

## 3. Discussion

Numerous surgical approaches have been developed to correct the webbed neck deformity seen in Noonan's and Turner's syndromes. The primary goals of correction include normalizing the appearance of the neck, raising the low hairline. Previously reported techniques have attempted correction from lateral or posterior approaches.

In 1937, Chandler originally proposed surgical correction of the deformity via bilateral Z-plasties [7]. A lateral approach allows for complete visualization of the web, but the use of a Z-plasty alone does not fully achieve desired aesthetic results. With this technique, the hair-bearing flap is rotated anteriorly and is visible on the lateral neck. Although the width of the neck is improved, future hair removal procedures would be indicated to achieve a normal appearance. Hikade et al. improved upon this technique by developing a modified Z-plasty [8]. With this approach a lateral Z-plasty is performed, the hair-bearing flap is excised, and the resultant dog-ear is corrected by two additional Z-plasties. This allows for visualization of the web and eliminates the need for future hair removal on the lateral neck. Although a modified Z-plasty does achieve the goals of webbed neck correction, scars extend from just below the mastoid process to the acromion.

With a posterior approach, excess skin is excised from midline of the posterior neck. This flattens the web against the lateral neck to achieve an improved appearance. Shearin and DeFranzo described a posterior butterfly correction of a webbed neck deformity in a Turner's patient [9]. Tension on the vertical limb of the scar caused considerable spreading and recurrence of the web. Similarly, Thomson et al. used a posterior approach and excised a midline ellipse of tissue [10]. The incision and resultant dog ears were closed leaving a Y-shaped scar. As was also described by Shearin and DeFranzo, the vertical limb of this scar is under considerable tension, which makes recurrence of the web possible [9].

Hypertrophic scarring was noted postoperatively in this case. A recent study noted that five out of eleven patients had mild to moderate postoperative hypertrophic scarring [11]. They found that lateral techniques with incorporation of Z-plasties provided superior results. They showed, as with our patient, that overall patient satisfaction is very high but recommended accurate preoperative planning and counseling of patients and families [11].

The M to T rearrangement described in this report offers considerable advantages to previously reported techniques. By using a lateral approach, the trapezial fascial web is directly visualized and able to be completely excised. This prevents the recurrence seen with the use of posterior techniques. Inferolateral displacement of hair-bearing skin can be removed with resection of the superior intervening triangle and improves the appearance of the low nuchal hairline. The excision of excess skin along with the zig-zag closure also prevents postoperative scar contraction and recurrence. An important effect of this technique is the prevention of anterior displacement of hair bearing skin. M to T rearrangement is a novel technique for effective surgical correction of webbed neck deformities seen in Noonan's and Turner's syndromes.

## References

[1] Noonan JA, Ehmke DA (1963). Associated noncardiac malformations in children with congenital heart disease. *Journal of Pediatrics*.

[2] Allanson JE (1987). Noonan syndrome. *Journal of Medical Genetics*.

[3] Tartaglia M, Mehler EL, Goldberg R (2001). Mutations in PTPN11, encoding the protein tyrosine phosphatase SHP-2, cause Noonan syndrome. *Nature Genetics*.

[4] Jongmans M, Sistermans EA, Rikken A (2005). Genotypic and phenotypic characterization of Noonan syndrome: new data and review of the literature. *American Journal of Medical Genetics*.

[5] Kobylinski O (1883). Ueber eine flughautähnliche austreitung am haise. *Am Hals Arch Anthoropol*.

[6] Funke F (1902). Pterygium colli. *Deutsche Zeitschrift für Chirurgie*.

[7] Chandler FA (1937). Webbed Neck (pterygium colli). *American Journal of Diseases of Children*.

[8] Hikade KR, Bitar GJ, Edgerton MT (2002). Modified Z-plasty repair of webbed neck deformity seen in Tuner and Klippel-Feil syndrome. *The Cleft Palate-Craniofacial Journal*.

[9] Shearin JC, DeFranzo AJ (1980). Butterfly correction of Webbed-Neck deformity in Turner’s syndrome. *Plastic and Reconstructive Surgery*.

[10] Thomson SJ, Tanner NSB, Mercer DM (1990). Web neck deformity; Anatomical considerations and options in surgical management. *British Journal of Plastic Surgery*.

[11] Reichenberger MA, Goertz O, Lehnhardt M (2013). Surgical correction of pterygium colli. *Journal of Pediatric Surgery*.

